# Mitochondrial Apoptotic Pathway Is Activated by H_2_O_2_-Mediated Oxidative Stress in BmN-SWU1 Cells from *Bombyx mori* Ovary

**DOI:** 10.1371/journal.pone.0134694

**Published:** 2015-07-30

**Authors:** Peng Chen, Yan-Fen Hu, La Wang, Wen-Fu Xiao, Xi-Yan Bao, Chun Pan, Hua-Shan Yi, Xiang-Yun Chen, Min-Hui Pan, Cheng Lu

**Affiliations:** 1 State Key Laboratory of Silkworm Genome Biology, Southwest University, Chongqing, 400716, China; 2 Key Laboratory for Sericulture Functional Genomics and Biotechnology of Agricultural Ministry, Southwest University, Chongqing, 400716, China; Institute of Plant Physiology and Ecology, CHINA

## Abstract

Apoptosis is a known regulator of morphogenetic events. In mammals, the critical role of oxidative stress-induced apoptosis has been well-studied; however, in insects the role of oxidative stress in apoptosis is not clear. In a previous study, we showed that apoptosis-related genes are present in the silkworm *Bombyx mori*, an important lepidopteran insect model. In this study, we evaluated the effect of H_2_O_2_-induced oxidative stress on apoptosis, reactive oxygen species (ROS) levels, mitochondrial response, cytochrome c release and apoptosis-related gene expression in the BmN-SWU1 cell line from *B*. *mori* ovaries. Our results showed that BmN-SWU1 cells exposed to H_2_O_2_ showed cell protuberances, cytoplasmic condensation, apoptotic bodies, DNA ladder formation and caspase activities indicating apoptosis. H_2_O_2_-induced apoptosis also increased intracellular ROS level, changed mitochondrial distribution, reduced mitochondrial membrane potential and increased the release of cytochrome c from mitochondria. Furthermore, western blot analysis revealed a significant increase in p53 and cytochrome c expression, and a decrease in Bcl-2 expression compared to the controls. Moreover, quantitative real-time PCR (qRT-PCR) showed an increase in the transcript levels of *BmICE*, *Bmapaf-1* and *BmEndoG* by 439.5%, 423.9% and 42.2%, respectively, after treatment with 1 μM H_2_O_2_ for 24 h. However, the transcript levels of *Bmbuffy* declined by 41.4% after 24 h of exposure to 1 μM H_2_O_2_. These results show that H_2_O_2_ treatment induced apoptosis in BmN-SWU1 cells via the mitochondrial apoptotic pathway. Further, it appears that oxidative stress induced by H_2_O_2_ activates both caspase-dependent and caspase-independent mitochondrial apoptotic pathways in silkworm cells. Taken together, these findings improve our knowledge of apoptosis in silkworm and the apoptotic pathways in insects.

## Introduction

Reactive oxygen species (ROS) has been implicated in a wide variety of cellular events such as proliferation, differentiation and programmed cell death [1–5]. In general, excess accumulation of ROS occurs due to a body’s inability to detoxify ROS thus resulting in oxidative stress. Within a cell, mitochondria are the major generators of ROS. It is now well recognized that ROS play a critical role in the activation of apoptotic process via the mitochondria-dependent and mitochondria-independent pathways [6–8]. However, the complex mechanisms underlying ROS-mediated programmed cell death or apoptosis are not well understood in insects.

Apoptosis is triggered by extrinsic and/or intrinsic signals thereby initiating a series of complex cellular pathways [9, 10]. The extrinsic pathway is activated by the binding of extracellular ligands to cell death receptors on the plasma membrane, while the intrinsic pathway is mainly induced by different mitochondrial stress factors [11, 12]. During apoptosis, cells undergo a series of morphological changes, such as formation of apoptotic bodies, chromatin condensation, blebbing of the membrane surface and DNA fragmentation [13]. The final effectors of apoptosis are the cysteine-dependent aspartate proteases or caspases [14] although caspase-independent pathways also exist. Recent reports have demonstrated that both caspase-independent and caspase-dependent pathways are involved in ROS-mediated apoptosis in some non-insect organisms [15, 16]. However, the mechanisms that regulate oxidative stress-induced apoptosis in insect cells are still poorly understood.

In the silkworm *Bombyx mori*, programmed cell death occurs extensively during development and metamorphosis due to remodeling of tissues and organs, such as the larval midgut, fat body and silk gland [17–19]. Although a number of apoptosis-related genes have been identified in the recently sequenced silkworm genome [20–23], the functional analysis of these proteins has not been performed extensively. A thorough understanding of the apoptotic pathways in *B*. *mori* will provide new insights and further our knowledge of apoptosis in this important lepidopteran model and other insects. Besides, studies on pathways involved in the H_2_O_2_-mediated oxidative stress in insects are limited. Therefore, we performed this study to determine the effects of H_2_O_2_-mediated oxidative stress on apoptotic signaling in an ovarian *B*. *mori* cell line, BmN-SWU1. Our results demonstrated that oxidative stress mediated by H_2_O_2_ induces BmN-SWU1 cell apoptosis via the mitochondrial-dependent pathway, and may activate both caspase-dependent and caspase-independent apoptotic pathways in these cells.

## Materials and Methods

### Cell culture and treatment

The *Bombyx mori* ovarian cell line, BmN-SWU1 [24], was cultured in TC-100 insect medium (USBiological, Swampscott, MA, USA) containing 10% (vol/vol) fetal bovine serum (FBS; PAA Laboratories) at 27°C. The cells were treated with various H_2_O_2_ concentrations (0, 0.1, 1.5, 10 μM) for 0, 8, 12, 16 or 24 h to establish the median lethal dose (LD_50_), defined as the dose at which cell viability is 50%.

### Cell viability assay and morphological observation

Cell viability was determined by MTT cell proliferation assay according to the manufacturer’s instructions (Beyotime, Jiangsu, China). Briefly, BmN-SWU1 cells were cultured in 96-well plates and treated with various H_2_O_2_ concentrations for 0, 8, 12, 16 or 24 h. Then, 10 μL of the MTT stock solution (5 mg/mL) was added to each well. After incubation for 4 h at 27°C, the MTT solution was removed and 100 μL formazan solution was added to each well. The cells were incubated for an additional 4 h at 27°C and the absorbance of the cell culture supernatant was measured at 570 nm using a microplate reader (Promega; Madison, WI, USA) to determine cell viability. Each assay was repeated three times.

Morphology of cells in culture was determined by fluorescence microscopy. First, H_2_O_2_ treated cells were fixed in 4% paraformaldehyde (PFA; Sangon, Shanghai, China) for 10 min at room temperature. After removal of the PFA solution the cells were washed with phosphate-buffered saline (PBS) three times for 5 min each. Then, PBS was removed and the cell nuclei were stained with DAPI (Beyotime) in the dark for 8 min. Finally, cell morphology was examined through a fluorescence microscope (Nikon, Tokyo, Japan) at an excitation wavelength of 360 nm.

### Flow cytometric analysis of apoptotic cells

Cell apoptosis was determined using an Annexin-V-FLUOS staining kit (Roche, Risch, Switzerland). In brief, the cells were first treated with 1 μM H_2_O_2_ for 0, 8, 12, 16 or 24 h and harvested by centrifugation in 5 mL centrifuge tubes at 800 g for 5 min. After washing once in PBS the cells were resuspended in 96 μL binding buffer (Roche), 2 μL Annexin-V-FLUOS and 2 μL propidium iodide (PI) at a final density of 1 × 10^6^ cells/mL and incubated for 15 min in dark at room temperature. Then, the cells were mixed with 200 μL binding buffer for analysis through flow cytometry (FACScan; Becton Dickenson, Oxford, UK).

### DNA Ladder

BmN-SWU1 cells treated for 24 h with 1 μM H_2_O_2_ were harvested to extract genomic DNA using the DNA Ladder Extraction Kit (Beyotime) according to the manufacture’s protocol. Then, the DNA were separated on a 1.5% agarose gel, stained with ethidium bromide and visualized under ultraviolet light.

### Measurement of caspase activity

Caspase activities were determined using the Caspase-Glo 9, 3/7 assay systems (Promega). Briefly, BmN-SWU1 cells were cultured in 96-well plates and treated with H2O2, as described above. The cells were incubated with 100 μL Caspase-Glo 9 (3/7) reagent for 3 h at room temperature in the dark and the fluorescence was measured using a microplate reader.

### Evaluation of intracellular ROS

A reactive oxygen species assay kit (Beyotime) was used to monitor the levels of intracellular ROS, according to the manufacturer’s protocol. BmN-SWU1 cells were seeded at equal densities in 96-well plates and treated with 1 μM H_2_O_2_ for various times. Then, they were incubated with 200 μL 2', 7'-dichlorodihydrofluorescein diacetate (DCFH-DA) (10 μM) at 37°C for 20 min in the dark. Control cells were not treated with DCFH-DA. After removal of DCFH-DA the cells were washed three times in serum-free cell culture medium to remove excess fluorescent probes. Fluorescence was then quantified using a fluorescence microplate reader at an excitation wavelength of 485 nm and emission wavelength of 528 nm. The increase in relative fluorescence indicated an increase in intracellular ROS levels.

### Measurement of mitochondrial membrane potential

A DiOC_2_(3) iodide (3,3’-diethyloxacarbocyanine iodide) assay kit (Invitrogen, Carlsbad, CA, USA) was used to evaluate mitochondrial membrane potential. Cells treated with H_2_O_2_ were harvested and resuspended in 1 mL warm culture medium at a density of 1 × 10^6^ cells/mL before incubation for 20 min at 37°C in 5 μL (10 μM) DiOC_2_(3) iodide. The cells were washed once in 2 mL warm PBS and resuspended in 200 μL PBS. DiOC_2_(3) iodide-stained cells were analyzed by flow cytometry. The mean mitochondrial membrane potential was evaluated by measuring the change in mean fluorescence intensity.

### Detecting mitochondrial distribution

Mito-Tracker Green (Molecular Probes; Eugene, OR, USA) was used to label mitochondria to observe the distribution of mitochondria within cells. First, BmN-SWU1 cells were cultured in 6-well plates and treated with H_2_O_2_ for different time periods. The cells were then incubated with 1 mL Mito-Tracker Green solution (100 nM) for 30 min at 37°C. The cells were washed twice with warm cell-culture medium before examination through a confocal microscope (Olympus, Tokyo, Japan) at an excitation wavelength of 488 nm. Green fluorescence reflected the mitochondrial distribution.

### Immunofluorescence detection of cytochrome c

BmN-SWU1 cells were seeded at equal densities and cultured on coverslips in a 24-well plate. They were then treated with H_2_O_2_ as described above, washed in PBS and fixed in 4% PFA for 15 min. Then, they were permeabilized with 1% Triton X-100 in PBS for 15 min at room temperature and blocked with 1% BSA and 10% goat serum in PBS for 1 h at 37°C. The cells were then incubated with mouse anti-cytochrome c primary antibody (1:100; Beyotime) for 2 h and then stained with Cy3-labeled goat anti-mouse secondary antibody (1:500; Beyotime) for 1 h at 37°C. The nuclei were stained by incubation with DAPI for 8 min in the dark, and then mitochondria were stained with 300 nM Mito-Tracker Green solution for 20 min at 37°C. Finally, the cells were visualized through a confocal microscope.

### Preparation of BmN-SWU1 cell cytosolic and mitochondrial fractions

BmN-SWU1 cells were treated with H_2_O_2_ for 12 h and harvested by centrifugation at 600 g for 5 min. The cells were washed once with cold PBS, resuspended in 500 μL lysis buffer (200 mM Hepes-KOH pH 7.5, 10 mM KCl, 1.5 mM MgCl_2_, 1 mM EDTA-Na_2_, 1 mM EGTA-Na_2_, 1 mM DTT, 0.1 mM PMSF, 250 mM sucrose) and placed on ice for 15 min. Then, they were homogenized in a glass homogenizer and centrifuged at 11,000 g for 10 min at 4°C. The supernatant was saved as the cytosolic fraction, and the pellet was resuspended in 200 μL lysis buffer and saved as the mitochondrial fraction.

### Quantitative real-time PCR (qRT-PCR)

The expression levels of apoptosis-related genes were evaluated by qRT-PCR. First, total RNA was extracted from H_2_O_2_-treated BmN-SWU1 cells using TRIzol Reagent (Invitrogen) according to the manufacturer’s instructions. Then, cDNA was synthesized in a 25 μL reaction mixture containing 2 μg total RNA, 2 μL Oligo dT, 2 μL dNTP, 1 μL M-MLV, 1 μL RNasin (Promega) and approximately 12 μL diethylpyrocarbonate (DEPC) water (Beyotime). The resulting cDNA was used in qRT-PCR (Step One Plus, Applied Biosystems, Carlsbad, CA, USA) to analyze the expression of apoptosis-related genes using gene specific primers. Each reaction was performed in a total volume of 15 μL using SYBR Green PCR reagents (TaKaRa, Dalian, China) and incubated for 4 min at 95°C, followed by 40 cycles of 95°C for 15 s and 60°C for 31 s, 95°C for 15 s, 60°C for 20 s and 95°C for 15 s. The ribosomal protein L3 (rpL3) was used as an internal control. Primers used in qRT-PCR are listed in Table 1.

**Table 1:** Primer sets used in this study.

Gene name	Forward primer (5'-3')	Reverse primer (5'-3')
*BmICE*	AGTATTCGCTGCCGACCAA	TAAGACGCCCCTGCTTCAC
*Bmapaf-1*	ACAGTTCACAACCCTCTAAAATCAC	CACTTTCTTACCACGCATCACC
*Bmbuffy*	TCAGCTATGCTACGCTCAGACA	ATCCATGATCCAGGCTCCTC
*BmEndoG*	CCGAGGAAAGAATCCGACG	CACCCACGACTACCTTGTAGAAAT
*BmrpL3*	CGGTGTTGTTGGATACATTGAG	GCTCATCCTGCCATTTCTTACT

### Western blot analysis

H_2_O_2_-treated BmN-SWU1 cells from each treatment were collected by centrifugation and added to radio-immunoprecipitation assay (RIPA) lysis buffer (Beyotime) on ice. After centrifugation at 13,000 g for 5 min at 4°C, the supernatants were extracted and mixed with sodium dodecyl sulfate (SDS) loading buffer. Total protein concentration was estimated using a bicinchoninic acid (BCA) Protein Assay Kit (Beyotime) as described by the manufacturer. Equal amounts of proteins were separated on a 10% SDS polyacrylamide gel electrophoresis (PAGE) and finally transferred onto polyvinylidene difluoride (PVDF) membranes using standard protocols. The membranes were then incubated with the following primary antibodies: anti-cytochrome c (1:500; Beyotime), anti-Bcl-2 (1:1000; Cell Signaling Technology, Danvers, MA, USA) and anti-P53 (1:1000; Cell Signaling Technology), followed by incubation with the corresponding horseradish peroxidase (HRP)-labeled secondary antibody (1:5000; Beyotime). Protein bands were visualized using the Lumi-Light PLUS Western blotting substrate (Roche, Mannheim, Germany). Tubulin (Beyotime) was used as the loading control.

## Results

### Induction of apoptosis by H_2_O_2_ in BmN-SWU1 cells

**Fig 1:**
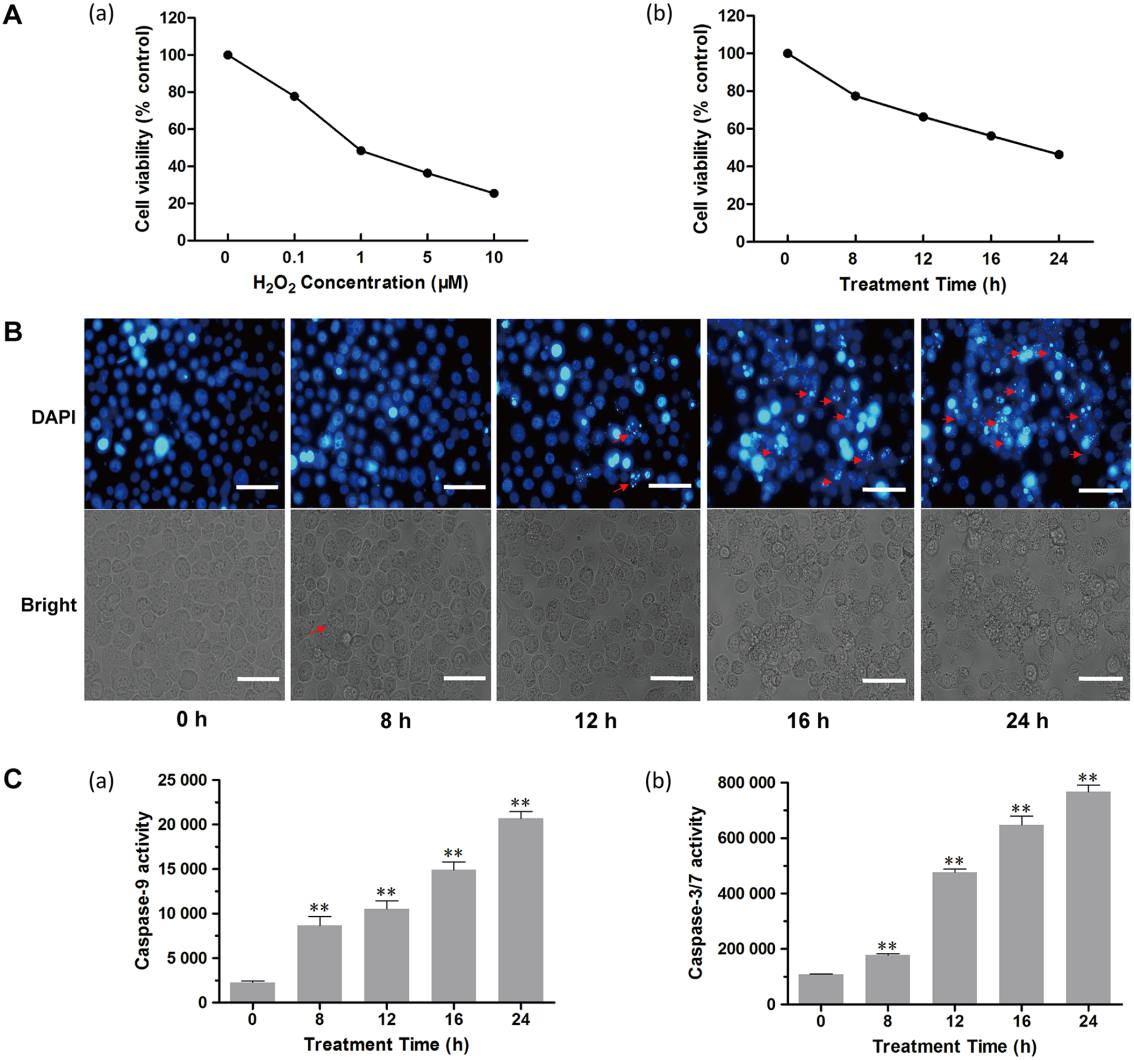
Apoptosis assays in BmN-SWU1 cells after exposure to H_2_O_2_. (A) Effects of H_2_O_2_ exposure on the viability of BmN-SWU1 cells treated with different concentrations of H_2_O_2_ for 24 h (a) and for different time periods with 1 μM H_2_O_2_ (b). (B) Fluorescence microscopic images of morphological changes in the nuclei of BmN-SWU1 cells treated with H_2_O_2_. Arrows indicate apoptotic bodies in the DAPI stained images (12–24 h), and the arrow in the bright field image (8 h) indicates the vesicles. Scale bars = 50 μm. (C) Effects of 1 μM H_2_O_2_ on caspase-9 (a) and caspase-3/7 (b) activities in BmN-SWU1 cells. All treated groups were compared with the control (0 h). Values represent mean ± SD (*n* = 3). **, *P* < 0.01, Student’s *t*-test.

In order to determine the optimal H_2_O_2_ required to induce apoptosis in BmN-SWU1 cells, we first treated the cells with various H_2_O_2_ concentrations (0, 0.1, 1.5, 10 μM) for different time points (0, 8, 12, 16, 24 h) and determined cell proliferation using the MTT assay. The results showed that H_2_O_2_ inhibited cell proliferation in a dose and time-dependent manner (Fig 1A). From these assays the median lethal dose (LD_50_) was estimated as 1 μM H_2_O_2_ with an exposure time of 24 h. Further, the morphology of BmN-SWU1 cells treated with H_2_O_2_ for 0, 8, 12, 16 and 24 h showed that the number of rounded and normal shaped cells was reduced after H_2_O_2_ treatment when compared to the control. In addition, when the treatment period was prolonged the number of abnormal cells increased, cell protrusions disappeared, and formation of vesicles and apoptotic bodies increased (Fig 1B). In addition, the effect of H_2_O_2_ on BmN-SWU1 cell apoptosis was also assessed by the Annexin V-FLUOS/PI flow cytometry and DNA ladder assays (S1 Fig). The results showed an increase in Annexin V staining and a longer smear of the DNA ladder in the H_2_O_2_ treated cells corroborating the morphological observations. Together, these findings indicate that H_2_O_2_ induces apoptosis in BmN-SWU1 cells.

Furthermore, we measured the protease activity of two important apoptotic signaling molecules, the initiator caspase-9 and effector caspase-3/7 in H_2_O_2_ treated BmN-SWU1 cells. The results demonstrated that both caspase-9 and caspase-3/7 activities increased significantly in a H_2_O_2_ treatment time-dependent manner. The maximum activities of caspase-9 and caspase-3/7 (Fig 1C) were observed after treatment for 24 h with a 9- and 7-fold increase, respectively when compared to the control cells (*P* < 0.01).

### Effects of H_2_O_2_ on intracellular ROS levels and mitochondria

**Fig 2:**
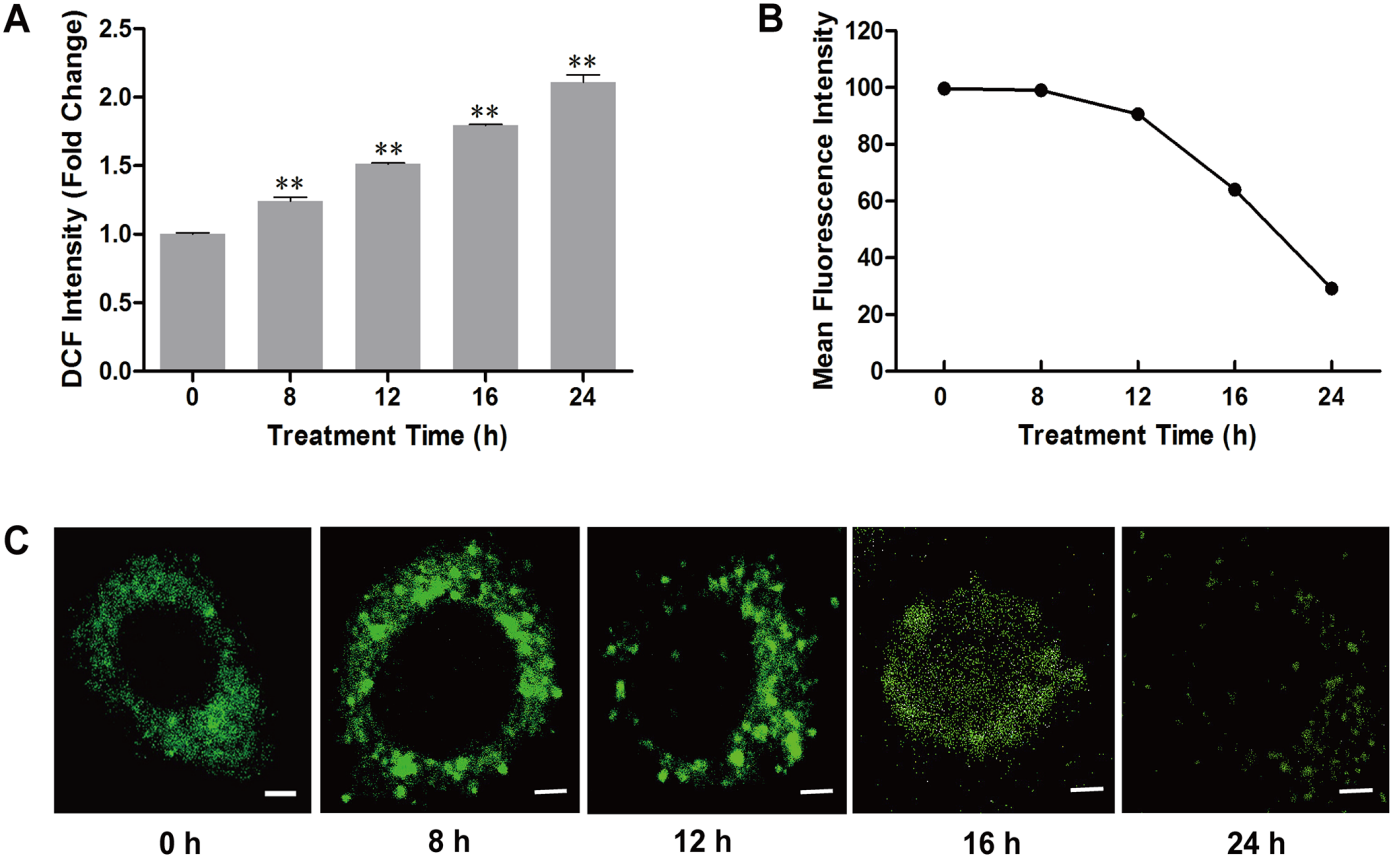
Effects of H_2_O_2_ on the intracellular ROS, membrane potential and mitochondrial distribution in BmN-SWU1 cells. (A) Accumulation of intracellular ROS in BmN-SWU1 cells after treatment with H_2_O_2_. All treated groups were compared with the control (0 h). Values are mean ± SD (*n* = 3). **, *P* < 0.01, Student’s *t*-test. (B) Effects of H_2_O_2_ exposure on the mitochondrial membrane potential (ΔΨm) in BmN-SWU1 cells. (C) Changes in mitochondrial distribution after H_2_O_2_ treatment. Scale bars = 5 μm.

H_2_O_2_ is a major component of ROS and has a strong ability for oxidation. In the present study, the level of ROS in H_2_O_2_ treated BmN-SWU1 cells was investigated by labelling with a DCFH-DA fluorescent probe. Our results showed that the DCF intensity gradually increased with treatment time reaching a maximum at 24 h suggesting that exposure of BmN-SWU1 cells to H_2_O_2_ resulted in a time-dependent increase in ROS levels (Fig 2A).

To further assess whether H_2_O_2_ could be involved in the mitochondrial-dependent apoptosis, we used a DiOC_2_(3) fluorescent probe to evaluate the changes in mitochondrial transmembrane potential (ΔΨm) by measuring the fluorescence intensity after exposure to H_2_O_2_. The mean fluorescence intensity was 99.7 in control BmN-SWU1 cells at 0 h. However, as the exposure time increased the mean intensity gradually decreased (Fig 2B and S2 Fig), indicating that H_2_O_2_ reduced the mitochondrial transmembrane potential in a time-dependent manner.

Subsequently, we detected changes in the mitochondrial distribution after treatment with H_2_O_2_. Confocal microscopy of BmN-SWU1 cells stained with Mito-Tracker Green revealed that the mitochondrial distribution was relatively homogeneous in the cytoplasm of untreated cells (Fig 2C). However, after treatment with H_2_O_2_ the homogeneous distribution of mitochondria was no longer apparent with aggregates visible at 8 h and 12 h post-treatment. At 16 h and 24 h post-treatment, the green fluorescence became more diffuse and the intensity decreased with increasing time (Fig 2C). These results suggested that H_2_O_2_ promoted changes in the distribution of mitochondria leading to mitochondrial depolarization.

### Expression and release of cytochrome c in H_2_O_2_-treated cells

**Fig 3:**
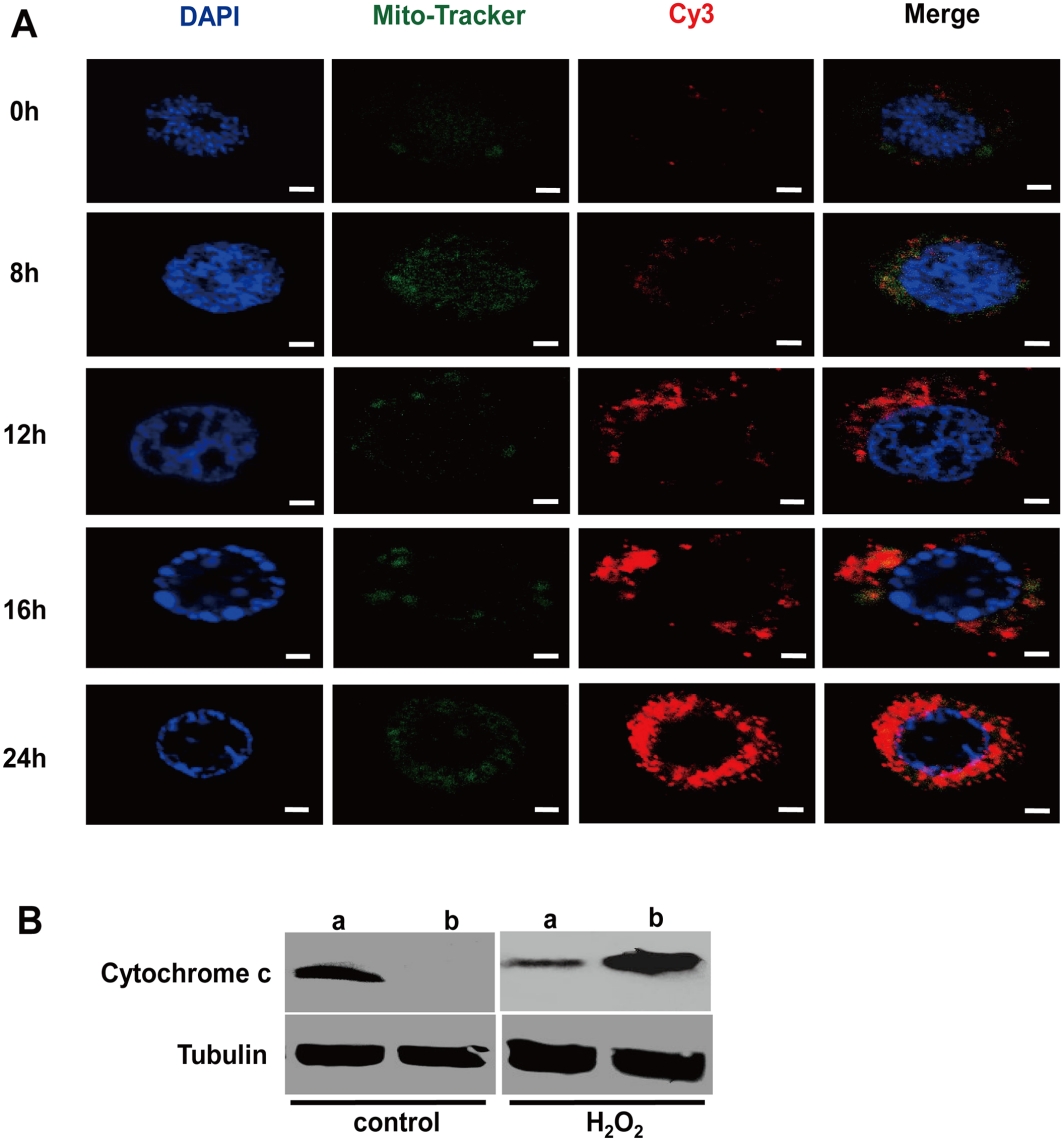
Changes in cytochrome c in BmN-SWU1 cells after exposure to H_2_O_2_. (A) Micrographs of BmN-SWU1 cells stained with DAPI (nuclei stained in blue), Mito-Tracker Green (mitochondria stained in green) and Cy3 (cytochrome c stained in red). Scale bars = 2 μm. (B) Western blot analysis of cytochrome c release from mitochondria into the cytosol in BmN-SWU1 cells treated with H_2_O_2_ for 12 h. Lanes (a) show mitochondrial cytochrome c; lanes (b) show cytosolic cytochrome c. Tubulin was used as an internal control.

Cytochrome c released from mitochondria has been proposed to be a key event that initiates apoptosis in mammals and some insects [25, 26]. To determine whether H_2_O_2_ can induce the expression of cytochrome c, we performed double immunofluorescence staining at different time points after treating BmN-SWU1 cells with 1 μM H_2_O_2_. The results showed no apparent difference in the levels of cytochrome c between the treatment and control groups at 8 h post-treatment. However, marked increases in cytochrome c were observed at 12, 16 and 24 h post-treatment (Fig 3A). In addition, western blot analysis also indicated that cytochrome c was released from the mitochondria into the cytoplasm 12 h post-treatment (Fig 3B). These results demonstrated that H_2_O_2_-mediated oxidative stress could induce the expression and release of cytochrome c in BmN-SWU1 cells.

### Expression levels of mitochondrial apoptosis-related genes

**Fig 4:**
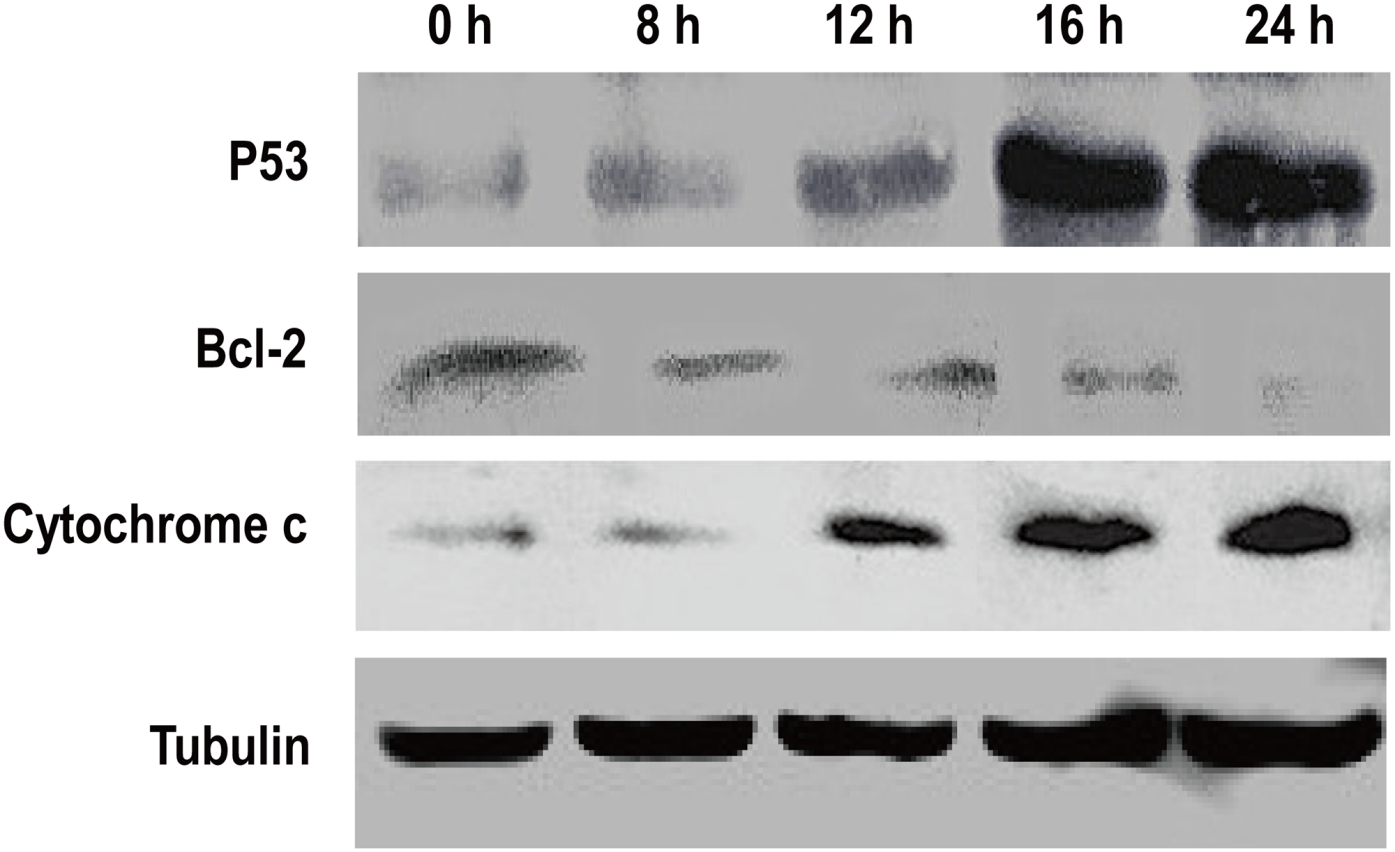
Effects of H_2_O_2_ exposure on the expression of apoptosis-related proteins in BmN-SWU1 cells. The band intensities reflect the expression levels of proteins after treatment with H_2_O_2_. Tubulin was used as an internal control.

Previous reports have demonstrated that p53, Bcl-2 and cytochrome c are associated with mitochondrial function and play important roles in cell apoptosis [27–30]. As shown in Fig 4, there was a clear increase in the levels of p53 and cytochrome c while the Bcl-2 protein levels decreased in a H_2_O_2_ treatment time-dependent manner. These results further suggested that H_2_O_2_ could induce apoptosis in BmN-SWU1 cells via the mitochondrial-dependent pathway.

**Fig 5:**
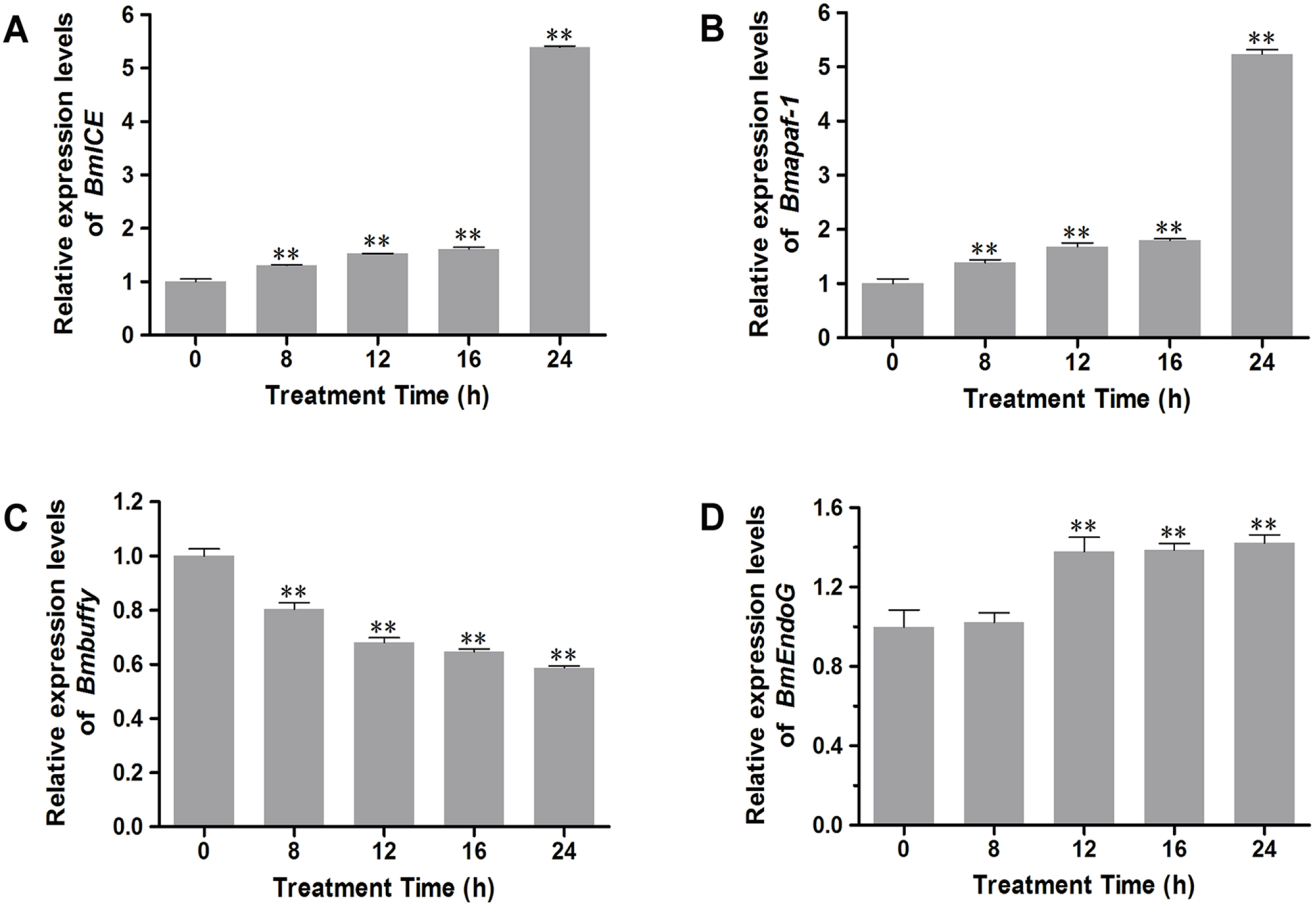
qRT-PCR analysis of apoptosis-related genes in BmN-SWU1 cells after exposure to H_2_O_2_. All treated groups were compared with the control (0 h). Values indicate mean ± SD (*n* = 3). **, *P* < 0.01, Student’s *t*-test.

Moreover, we investigated the mRNA levels of four other apoptosis-related genes to determine whether apoptosis induced by H_2_O_2_ in BmN-SWU1 cells is through the caspase-dependent or caspase-independent pathway. We first examined the expression levels of *BmICE*, *Bmapaf-1* and *Bmbuffy* involved in the caspase-dependent apoptosis. We found that there was a 439.5% and 423.9% increase in the *BmICE* and *Bmapaf-1* mRNA expression, respectively after exposure to 1 μM H_2_O_2_ for 24 h (Fig 5A and 5B). On the other hand, *Bmbuffy* expression decreased by 41.4% after 24 h treatment with 1 μM H_2_O_2_ (Fig 5C). Next, we evaluated the expression level of *B*. *mori endonuclease G* (*BmEndoG*), which is implicated in the caspase-independent apoptotic pathway in the mitochondria [31]. The results showed that there was a 42.2% increase after exposure of BmN-SWU1 cells to 1 μM H_2_O_2_ from 12–24 h (Fig 5D) indicating that oxidative stress induced by H_2_O_2_ may also activate caspase-independent apoptotic pathway in BmN-SWU1 cells.

## Discussion

Apoptosis is a conserved mechanism that is critical particularly during the morphogenetic events of an organism and has been well-studied in mammals. However, the mechanisms of apoptosis in insects are not well understood. In a previous study, we showed that in *B*. *mori* both intrinsic and extrinsic apoptosis pathways may exist [21]. In the present study, we evaluated the effects of H_2_O_2_-mediated oxidative stress on apoptosis induction, ROS levels, mitochondria and apoptosis-related genes in BmN-SWU1 cells from the silkworm ovary. Our findings showed that exposure to H_2_O_2_ induced classical apoptosis related morphological and cellular changes, and lead to aberrant mitochondrial distribution, reduced mitochondrial membrane potential, release of cytochrome c and changes in caspase-9 and caspase-3/7 activities (Figs 1–3). These results are evidence for the involvement of a mitochondrial pathway in the H_2_O_2_-induced apoptotic process in BmN-SWU1 cells. Moreover, these results suggested that the mitochondrial apoptotic pathways in BmN-SWU1 cells are associated with the release of cytochrome c, which is different from *Drosophila* BG2 cells in which cytochrome c is not released from mitochondria [32]. More importantly, H_2_O_2_ affected the expression of genes and proteins involved in both caspase-dependent and caspase-independent mitochondrial apoptotic pathways suggesting that oxidative stress induced by H_2_O_2_ may activate both pathways in BmN-SWU1 cells.

Mitochondria are essential for the intrinsic apoptosis pathway and it has been shown that the dysfunction of mitochondria leads to apoptotic cell death [33]. A number of studies have demonstrated that mitochondrial proteins, such as cytochrome c can trigger caspase-dependent apoptosis [34]. In our study, cytochrome c was also released from the mitochondria into the cytoplasm in a time-dependent manner (Figs 3 and 4) suggesting that oxidative stress induced by H_2_O_2_ may activate the caspase-dependent apoptotic pathway. In addition, we found that Bmp53 level was markedly elevated but BmBcl-2 levels decreased after exposure to H_2_O_2_ (Fig 4). This result is also consistent with our previous report that Bmp53 may contribute to the release of cytochrome c in *B*. *mori* [22]. In mammals, studies have reported the involvement of Bcl-2 family of genes and *apaf-1* in the mitochondrial apoptotic event [30, 35]. In silkworm, *Bmbuffy* gene has been identified as an anti-apoptotic gene belonging to the Bcl-2 family [22], and it was speculated that *Bmapaf-1* also plays a pivotal role in apoptotic pathway [21]. In this study, the qRT-PCR results showed that H_2_O_2_ can inhibit *Bmbuffy* expression, and promote the expression of *Bmapaf-1* and *BmICE*, an executioner of apoptotic caspase (Fig 5A–5C). These findings further indicate that the caspase-dependent mitochondrial apoptotic pathway was involved in BmN-SWU1 cell apoptosis induced by H_2_O_2_.

We also examined the expression of *BmEndoG*, a homolog of *endonuclease G*, involved in caspase-independent apoptosis in mammals [36]. The result from the present study showed a marked increase in *BmEndoG* mRNA expression after exposure to H_2_O_2_ for 12, 16 and 24 h compared to the control (Fig 5D) indicating that oxidative stress induced by H_2_O_2_ may also activate caspase-independent mitochondrial apoptotic pathway. Further studies are needed to evaluate the contributions of these genes to the mitochondrial apoptotic signaling pathways in insects.

Interestingly the mitochondrial membrane potential remained unchanged after H_2_O_2_ treatment for 8 h (Fig 2B), whereas the caspase activity registered a marked change (Fig 1C). Findings of previous studies on the mitochondrial membrane potential have shown conflicting results; while in some studies the mitochondrial membrane potential declined in response to apoptotic stimuli [37–39], other studies observed no changes in membrane potential until cytochrome c was released [40, 41]. In the present study, the time of mitochondrial membrane potential decreased was consistent with cytochrome c release around 16 h (Figs 2B and 3A). Another explanation could be the presence of higher amounts of Bcl-2 (at 0 and 8h) (Fig 4), which has been reported as a potent inhibitor of apoptotic cell death [27, 30]. Accordingly, it is presumable that Bcl-2 may inhibit the initial decrease in mitochondrial membrane potential in BmN-SWU1 cells, and similar observations were also reported by other studies [38, 42].

## Supporting Information

S1 Fig Flow cytometry and DNA ladder analysis. (A) Flow cytometry of apoptosis in BmN-SWU1 cells after exposure to H_2_O_2_. (B) DNA Ladder based evaluation of apoptosis in BmN-SWU1 cells following exposure to H_2_O_2_. M, Marker; Lane 1, control (0 h); Lane 2, BmN-SWU1 cells treated with 1 μM H_2_O_2_ for 24 h.
(TIF)

S2 Fig Changes in the mitochondrial membrane potential in BmN-SWU1 cells exposed to H_2_O_2_ by flow cytometry. (TIF)
