# Supplementary figures and images for: Mitochondrial Apoptotic Pathway Is Activated by H2O2-Mediated Oxidative Stress in BmN-SWU1 Cells from Bombyx mori Ovary
Source: PLoS One. 2015 Jul 30;10(7):e0134694. doi: 10.1371/journal.pone.0134694 (PMC4520666; doi:10.1371/journal.pone.0134694)

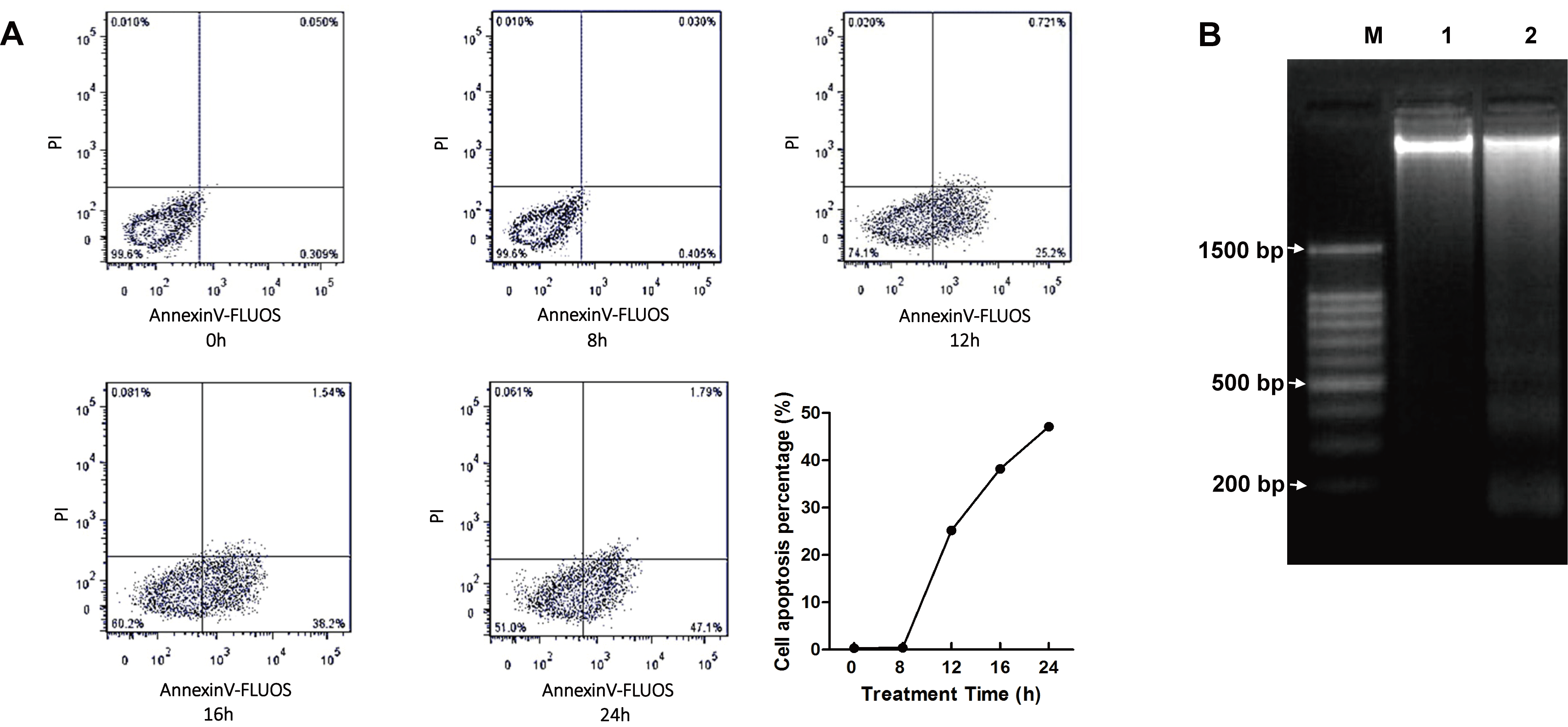

Supplement: S1 Fig — (A) Flow cytometry of apoptosis in BmN-SWU1 cells after exposure to H2O2. (B) DNA Ladder based evaluation of apoptosis in BmN-SWU1 cells following exposure to H2O2. M, Marker; Lane 1, control (0 h); Lane 2, BmN-SWU1 cells treated with 1 μM H2O2 for 24 h. (TIF) [file pone.0134694.s001.tif]

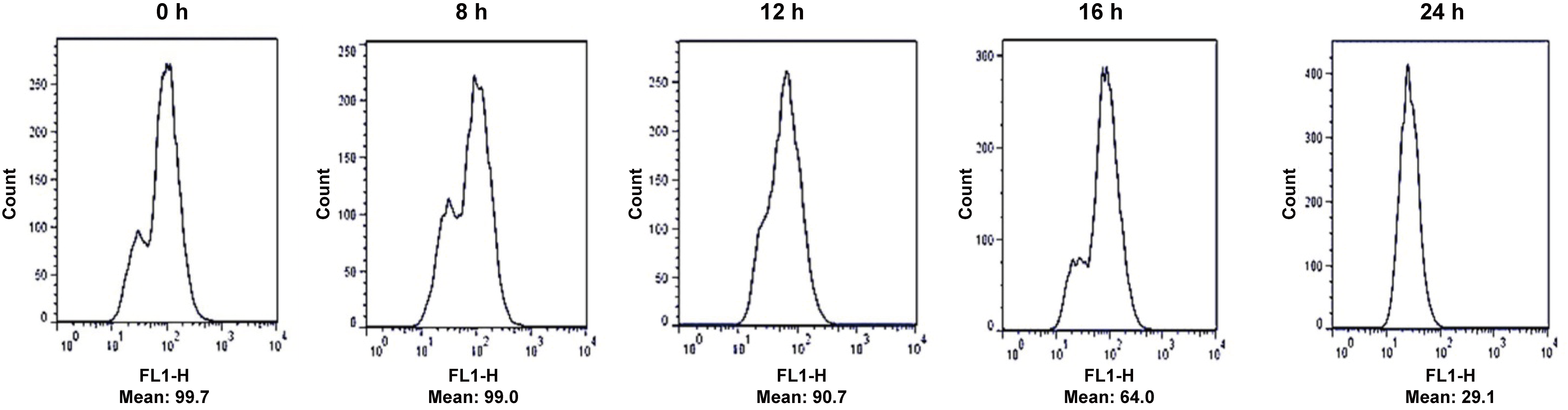

Supplement: S2 Fig — (TIF) [file pone.0134694.s002.tif]
